# How to measure autobiographical amnesia: time to upgrade the CUAMI?

**DOI:** 10.1192/j.eurpsy.2023.93

**Published:** 2023-07-19

**Authors:** B. Isidoor

**Affiliations:** Amsterdam UMC, Amsterdam, Netherlands

## Abstract

**Abstract:**

Autobiographical memory loss is a common complaint of patients treated with electroconvulsive therapy (ECT). However, reliable measurement of autobiographical memory loss after ECT is still a subject of debate. In the 1990s, the Colombia University Autobiographical Memory Interview (CUAMI) was developed specifically to monitor changes in autobiographical memory during a course of ECT. However, this instrument has been criticized due to the lack of psychometric data. In this talk, we will discuss the experiences of administrators and preliminary results of a study into the psychometric properties of the CUAMI. Is the CUAMI useful to test autobiographical memory after ECT or could it use an upgrade?

**Disclosure of Interest:**

B. Isidoor Grant / Research support from: Isidoor Bergfeld works on a clinical trial of DBS for depression sponsored by Boston Scientific (in kind). He also received grants from ZonMw and Amsterdam Brain and Cognition for studies on DBS and ECT

